# The complete chloroplast genome of *Prinsepia uniflora* (Rosaceae), a medicinal plant found in northwest China

**DOI:** 10.1080/23802359.2021.1978892

**Published:** 2021-09-24

**Authors:** Yali Wang, Yuan Niu, Zhu Qiang, Ying Tian, Yi Li

**Affiliations:** a College of Forestry, Gansu Agriculture University, Lanzhou, Gansu, China; b State Key Laboratory of Seedling Bioengineering, Ningxia Forestry Institute, Yinchuan, Ningxia, China; c College of Horticulture, Gansu Agricultural University, Lanzhou, Gansu, China; d Lanzhou Agro-technical Research and Popularization Center, Lanzhou, Gansu, China

**Keywords:** *Prinsepia uniflora*, chloroplast genome, Illumina sequencing, phylogenetic analysis

## Abstract

*Prinsepia uniflora* Batalin 1892 is a medicinal plant widely distributed in northwest China. In this study, we report and characterize the complete chloroplast (cp) genome sequence of *P. uniflora*. The entire sequence is 159,179 bp in length, consisting of the large single-copy region (LSC) and small single copy region (SSC) (87,239 and 19,180 bp, respectively); these two regions are separated by a pair of 26,380-bp inverted repeat (IR) regions. The genome contains 131 genes, including 86 protein-coding genes, 37 tRNA genes, and eight rRNA genes. The overall GC content of the genome is 36.7%. A phylogenetic tree constructed from 18 chloroplast genomes revealed that *P. uniflora* was clustered with *Prinsepia sinensis* and *Prinsepia utilis,* all of which belong to the genus *Prinsepia,* which is supported as a sister group by a moderate bootstrap support value of 55% with the *Malus* and *Pyrus* genera.

*Prinsepia uniflora*—a deciduous shrub—belongs to the genus *Prinsepia* (Rosaceae). It is a classic traditional Chinese medicinal plant (Zhang 2011; Li et al. 2012; Liu et al. 2021) and is mainly distributed in northwest China (Yang et al. 2008; Wang et al. 2012). The kernel of this plant, which is called ‘ruiren’ in traditional Chinese medicine, has long been used for the treatment of eye diseases (Zhou et al. 2013; Ma et al. 2020). Despite the importance of this plant, there is little information on the genome of *P. uniflora*. Here, we characterized the complete chloroplast (cp) genome sequence of *P. uniflora* based on genome skimming sequencing data to provide a valuable complete cp genomic resource.

Total genomic DNA was extracted from fresh leaves of *P. uniflora* grown in Yinchuan Botanical Garden (Yinchuan, Ningxia, China, 38°25′ N, 106°10′ E) using a modified cetyltrimethylammonium bromide (CTAB) method (Doyle and Doyle 1987). A specimen was deposited at the Herbarium of Yinchuan Botanical Garden (www.nxaas.com.cn; contact person: Zhu Qiang, email: qzhu2008@163.com) under the voucher number RH1025; DNA (NFI-20210517RH01) was kept at the State Key Laboratory of Seeding Bioengineering, Ningxia Forestry Institute. Whole-genome sequencing was conducted using the Novaseq 6000 platform (Illumina, USA) (Genepioneer Biotechnologies Co. Ltd., Nanjing, Jiangsu, China). The filtered sequences were assembled using SPAdes assembler 3.10.1 (Bankevich et al. 2012). Annotation was performed using DOGMA (Wyman et al. 2004) and BLAST searches. A physical map was generated using OGDRAW (Greiner et al. 2019). A phylogenetic tree was inferred based on the maximum likelihood using RAxML v8.2.10 (Stamatakis 2014).

The whole cp genome of *P. uniflora* (GenBank accession number: MZ270554.) is 159,179 bp in length, consisting of two inverted repeats (IRa and IRb) regions of 26,380 bp, a large single copy (LSC) region of 87,239 bp, and a small single-copy (SSC) region of 19,180 bp. The cp sequence contains 131 complete genes, including 86 protein-coding genes, 37 tRNA genes, and eight rRNA genes. Intron-exon structure analysis indicated that the majority (108) of genes have no introns, whereas 19 genes have a single intron and four protein-coding genes contain two introns. The overall GC content of the cp genome is 36.65%, while the corresponding values in the LSC, SSC, and IR regions are 34.44, 30.16, and 42.66%, respectively.

To further clarify the phylogenetic characteristics of *P. uniflora*, alignment was performed on the 18 complete chloroplast genome sequences (17 *Rosaceae* chloroplast genomes and 1 *Glycine soja* genome as outgroup) using MAFFT v7.427 (Katoh and Standley 2013). Phylogenetic analysis (Figure 1) showed that *P. uniflora* was first clustered with *P. sinensis* and then with *P. utilis*, all of which belonged to the genus *Prinsepia.* Genus *Prinsepia* is supported as a sister group with the *Malus* and *Pyrus* genera with a moderate bootstrap support value of 55%. Nine species of the genus *Prunus* clustered together. The species *Sorbaria arborea* clustered together with the genus *Prunus* (bootstrap value 73%).*Rubus eucalyptus* is a separated clade. The complete cp genome sequence of *P. uniflora* provides useful information for conservation genetics and phylogenetic studies of this species.

**Figure 1.**
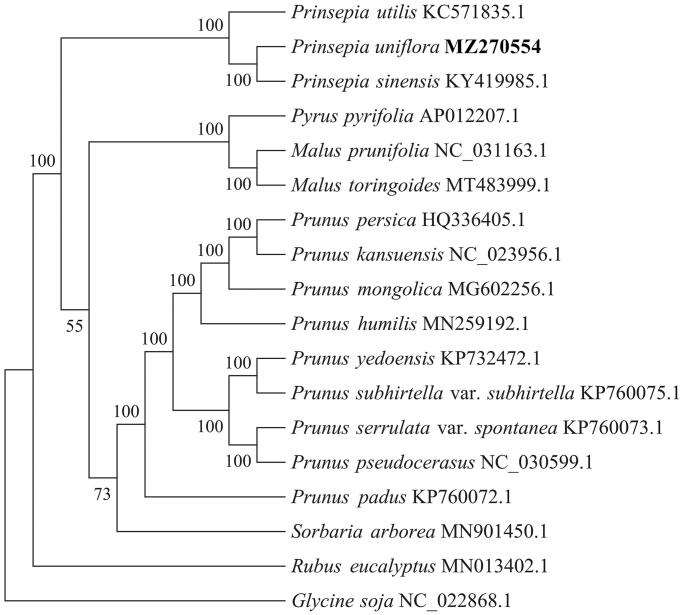
Phylogenetic tree inferred using maximum-likelihood (ML) method based on the complete chloroplast genome of 18 representative species, including 17 Rosaceae chloroplast genomes and 1 Glycine soja genome as the outgroup. Bootstrap values are shown as percentages at the branches. The complete chloroplast genome is downloaded from the NCBI database and shown after each species name.

## Data Availability

The genome sequence data that support the findings of this study are openly available in GenBank of NCBI at https://www.ncbi.nlm.nih.gov/ under the accession no. MZ270554. The associated BioProject, SRA, and Bio-Sample numbers are PRJNA757175, SRR15573078, and SAMN20938800, respectively.
